# František Nábělek’s Iter Turcico-Persicum 1909–1910 – database and digitized herbarium collection

**DOI:** 10.3897/phytokeys.75.9780

**Published:** 2016-12-01

**Authors:** Matúš Kempa, John Edmondson, Hans Walter Lack, Janka Smatanová, Karol Marhold

**Affiliations:** 1 Institute of Botany, Slovak Academy of Sciences, Dúbravská cesta 9, SK-845 23 Bratislava, Slovak Republic; 2 Royal Botanic Gardens, Kew, Richmond, Surrey, TW9 3AE, U.K.; 3 Botanischer Garten und Botanisches Museum Berlin-Dahlem, Freie Universität Berlin, Königin-Luise-Strasse 6-8, D-14195 Berlin, Germany; 4 Department of Botany, Faculty of Science, Charles University in Prague, Benátská 2, CZ-128 01 Praha 2, Czech Republic

**Keywords:** Database, Herbarium, Near East, František Nábělek, Vascular Plants

## Abstract

The Czech botanist František Nábělek (1884−1965) explored the Middle East in 1909-1910, visiting what are now Israel, Palestine, Jordan, Syria, Lebanon, Iraq, Bahrain, Iran and Turkey. He described four new genera, 78 species, 69 varieties and 38 forms of vascular plants, most of these in his work *Iter Turcico-Persicum* (1923−1929). The main herbarium collection of Iter Turcico-Persicum comprises 4163 collection numbers (some with duplicates), altogether 6465 specimens. It is currently deposited in the herbarium SAV. In addition, some fragments and duplicates are found in B, E, W and WU. The whole collection at SAV was recently digitized and both images and metadata are available via web portal www.nabelek.sav.sk, and through JSTOR Global Plants and the Biological Collection Access Service. Most localities were georeferenced and the web portal provides a mapping facility. Annotation of specimens is available via the AnnoSys facility. For each specimen a CETAF stable identifier is provided enabling the correct reference to the image and metadata.

## Introduction

**Figure 1.**
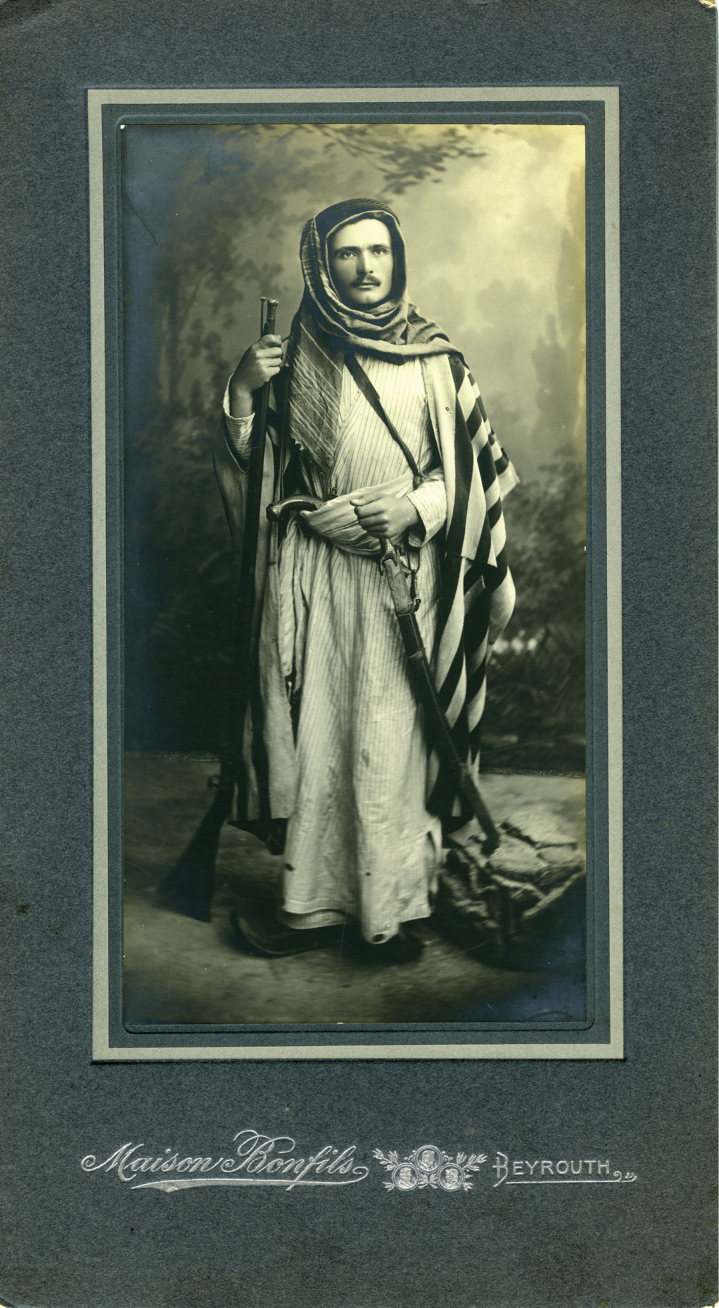
František Nábělek during his travels in 1909–1910 (photo courtesy of family Nábělek).

**Figure 2.**
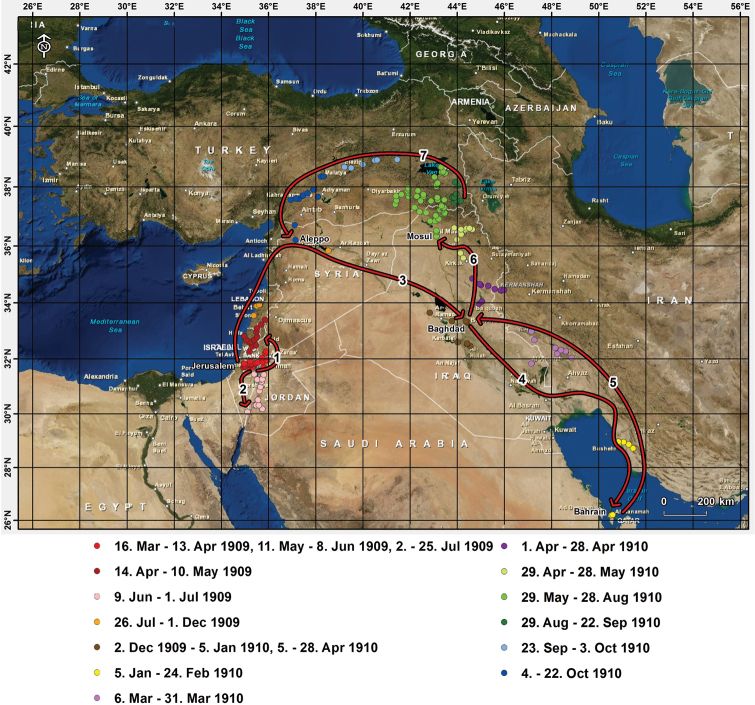
Schematic presentation of Nábělek's Iter Turcico-Persicum.

František Nábělek was born on 3 May 1884 in Kroměříž. From 1902 to 1907 he studied at Vienna University (Fig. 1, for further biographic details on František Nábělek see Table 1). With the financial support of the Imperial Royal Ministry of Culture and Education, the Imperial Academy of Sciences (both Vienna), the Svatobor Foundation (Prague) and the archbishop of Olomouc František Saleský Bauer in 1909-1910 Nábělek visited the region comprising the current Israel, Palestine, Jordan, Syria, Lebanon, Iraq, Bahrain, Iran and Turkey (Fig. 2). His plant collecting activities were among the most productive to have taken place in this region in the ﬁrst decade of the 20th century, and his subsequent publications (*Iter Turcico-Persicum*, Partes I-V, Nábělek 1923–1929, Nábělek 1922, 1924) included descriptions of four new genera, 78 species, 69 varieties and 38 forms of vascular plants. When working on plant identifications and descriptions of new taxa he extensively consulted other herbarium collections, particularly those of Pierre Edmond Boissier and Augustin Pyramus de Candolle in Geneva, Switzerland (G) and of Herbarium Haussknecht at that time held in Weimar, Grand Duchy of Saxe-Weimar-Eisenach, German Empire (now deposited in JE). He was also in contact with numerous specialists working on the flora of the Near East (such as Joseph Bornmüller, Weimar and Heinrich Handel-Mazzetti, Vienna). On the study of material of the genus *Onobrychis* Mill. (Fabaceae) he cooperated with Grigorij Ivanovič Širjaev of Masaryk University, Brno. There is extensive correspondence he sent to his family during his 1909-1910 journey with a number of details on his travels (in Czech) in the archives of the Institute of Botany, Slovak Academy of Sciences, Bratislava. All these letters have been recently digitized. Some notes relevant to his travels are also kept at the Central Archive of the Slovak Academy of Sciences. They are, however, not easy to read as they are mostly written in stenographic (shorthand) writing. After holding the position at the Masaryk University in Brno (now Czech Republic) in 1939 Nábělek moved to Bratislava (now in the Slovak Republic), where he organized the Institute of Botany at the newly established Faculty of Science. Since 1950, as Czechoslovakia had fallen under the Soviet sphere of inﬂuence, he was for political reasons no longer *persona grata* at the University and in order not to have any influence on students, he was forced to retire. Nevertheless, later he continued to work in the Arboretum in Mlyňany, where he established an herbarium collection of mostly cultivated exotic trees (MLY, in 2006 transferred to SAV as SAV-MLY) and prepared a complete botanical inventory of the Arboretum (Nábělek 1958). František Nábělek died on 10 June 1965 in Uherské Hradiště (now Czech Republic). His successor at Comenius University in Bratislava, Jozef Májovský, characterized him as “… an excellent teacher, organiser, musician and a good man. The Slovakian botanists will forever be grateful to him …” (Májovský 1966). More detailed biographical data on František Nábělek can be found in Hrabětová (1954, 1966), Hrabětová-Uhrová (1964), Májovský (1966, 1967), Michalko (1966), Stafleu and Cowan (1981: 678–679), Marhold and Feráková (1993) and Hrabovec (2010).

**Table 1.** Biographic data of František Nábělek (more details in Hrabětová 1954, 1964, Hrabětová-Uhrová 1964, Májovský 1966, 1967, Michalko 1966, Stafleu and Cowan 1981: 678–679, Marhold and Feráková 1993, Vacek and Bureš 2001, and Hrabovec 2010).

3 May 1884	Born (http://actapublica.eu/matriky/brno/prohlizec/8997/?strana=129) in Kroměříž, at that time in Austro-Hungarian Empire, now the Czech Republic
1902–1907	Studies at Vienna University under Professor Richard von Wettstein, who held a post at the University of Prague prior to being appointed as Director of the Botanical Gardens and Botanical Institute of the University of Vienna, now Austria
1907–1921	Secondary school (gymnasium) teacher in Lipník nad Bečvou, now Czech Republic
1909–1910	Iter Turcico-Persicum (for details see in the Introduction)
1921–1939	Secondary school (gymnasium) teacher in Brno, now Czech Republic
1925–1934	Private Associated Professor of Botany at the Masaryk University in Brno (now Czech Republic)
1934–1939	Extraordinary Professor of Botany at the Masaryk University in Brno (now Czech Republic)
1939	Lecturer at the Faculty of Philosophy of the Slovak University (before 1939 and after 1954 Comenius University), Bratislava, now Slovak Republic
1940	Professor of Botany and a chair at the Institute of Botany of the newly established Faculty of Science of the Slovak University, Bratislava, now Slovak Republic
1942–1950	Director of the University Botanical Garden of the Slovak University, Bratislava, now Slovak Republic
1945–1947	Dean of the Faculty of Science of the Slovak University, Bratislava, now Slovak Republic
1947–1948	Rector of the Slovak University, Bratislava, now Slovak Republic
1950	Forced retirement from the Slovak University, Bratislava, now Slovak Republic
1950–1953	Institute of Food Industry, Bratislava, now Slovak Republic
1953–1960	Researcher at Arboretum in Mlyňany, now Slovak Republic. The Arboretum Mlyňany (Benčať et al. 1956), was originally a private estate established in 1892 by Štefan (István) Ambrózy-Migazzi (1869, Nice, France – 1933, Tana, now Tanakajd, Hungary), later becoming a state-funded scientiﬁc institute of the Slovak University, before its final transfer to the Slovak Academy of Sciences in 1953.
10 June 1965	Died in Uherské Hradiště (now Czech Republic), buried in Bratislava at Slávičie údolie Cemetery

Here we present detailed data on Nábělek’s Iter Turcico-Persicum, together with an on-line database with digitized and georeferenced specimens and their metadata.

## The itinerary

The primary source of information for the itinerary of Iter Turcico-Persicum is Nábělek’s own published itinerary (Nábělek 1923: 3−12). However, due to the considerable problems of recording and transliterating place-names from a plethora of languages, further complicated by the “ethnic cleansing” of indigenous place-names in certain areas and their replacement by made-up modern names in the national languages, botanists wishing to trace the localities visited by Nábělek and determine their location and modern equivalents have encountered considerable obstacles. For a detailed discussion of these problems, the reader is referred to a useful publication of the United States Board on Geographic Names – Foreign Names Committee (2015). Although this report covers Iran, it does embrace most of the local languages encountered by Nábělek with the possible exception of Armenian. A further source for ethnic minority place names for localities in Turkey is represented by Index Anatolicus (Nişanyan 2014).

The primary method adopted in tracing localities has been to “ﬂy” Nábělek’s route using GoogleEarth, ﬁrst pinpointing known locations and then examining possible routes to see whether intermediate place-names could be matched with the spellings adopted by Nábělek. In the case of certain mountains, altitudes cited by Nábělek have provided further evidence.

In many instances, botanists citing specimens collected by Nábělek have already determined the modern equivalents of his collecting sites, and this information has been incorporated into our gazetteer. For example, on 17 June 1910 he visited Hašîtha in southernmost Turkey; this is a Kurdish place-name (now written Aşutka) which on modern maps has been replaced with the Turkish name Çığlı. A Persian locality, Nábělek's Chôšab (now written Hoşap) which he visited on 29 August 1910 has been re-named Güzelsu in Turkish (which signiﬁes ‘beautiful water’ in both languages).

In the earlier part of his travels, Nábělek seems to have chosen a route that would allow him to visit famous (e.g., Petra) and not-so-famous (e.g., Ain ez-Zarra, the ancient Callirhoe hot springs on the eastern bank of the Dead Sea) historic sites. Later, when travelling in northern Iraq, he seems to have sought out Nestorian Christian monasteries. Because those establishments were named using ancient languages such as Syriac, they are often unrecognisable on modern maps.

His journey was interrupted by two lengthy spells of illness, the ﬁrst from August to November 1909 in Damascus and Beirut (recuperating in the hilly suburb of Brumana) and the second in August 1910 which he spent in the eastern Anatolian town of Van. Despite these setbacks he managed to explore large areas of mountainous terrain in northeastern Iraq and the remotest parts of southeastern Turkey, areas previously only poorly explored and with a high level of ﬂoristic endemism, providing him with a rich haul of new species.

During his travels Nábělek must have encountered some interesting companions. Perhaps the most famous of these was the German Vice-Consul in Bushehr, Wilhelm Wassmuss, “the German Lawrence” (von Mikusch 1937 [other references at https://de.wikipedia.org/wiki/Wilhelm_Wassmuss]), with whom he ascended “Kuh Chormûǧ” (Kuh-e Khormuj, c. 1870 m), a prominent hill in Iranian Khuzestan. This expedition took six days, suggesting that it was achieved on foot. Wassmuss was only four years older than Nábělek, who had celebrated his 26^th^ birthday three months after the start of the excursion on 15 February, and since Wassmuss returned to Iraq later that same year it was highly fortuitous that they met during this brief interval in Iran. Later in his travels, on 10 March 1910, Nábělek visited Shushtar, a town only some 40 km from Masjed Soleiman, where oil had ﬁrst been discovered just a year earlier.

## Collection

The main herbarium collection of František Nábělek’s Iter Turcico-Persicum currently comprises 4163 collection numbers; altogether 6465 specimens (including duplicates). Only a few specimens have been lost during the many transfers between herbaria and loans in the past. The collection was first deposited in the herbarium of the Masaryk University in Brno (currently Czech Republic, BRNU) at the time of Nábělek’s professorship at this University. Then it was moved to University in Bratislava, Slovakia (currently herbarium SLO). After Nábělek left the Slovak (now Comenius) University, the herbarium was first kept in Arboretum Mlyňany, Slovakia (MLY), then at the Institute of Botany of the Slovak Academy of Sciences, Bratislava (acronym originally BAV, now SAV) and for some time also in the Slovak National Museum in Bratislava (BRA). The final place of deposit of this collection is the herbarium SAV. As a result, Nábělek’s type specimens are often referred to in the literature as deposited in BRNU (e.g., Stafleu and Cowan 1981), BRA, SLO or BAV, although they are now all held in SAV. Only some fragments and duplicates are found in B, E, W and WU. Indeed, in some cases they represent original material and may serve as types.

## Digitization

With the financial support of the Andrew W. Mellon Foundation, the whole of Nábělek’s Iter Turcico-Persicum collection at SAV was recently digitized. The process of digitization was performed in compliance with the JSTOR Plant Science Handbook (http://about.jstor.org/content/jstor-plant-science-handbook-english). All specimens were barcoded with a unique barcode within the institution before the scanning of the herbarium sheets. The barcode consists of letters ‘SAV’ followed by 7 digits. An Epson Expression Model 10000XL scanner was used for scanning, which was placed on a custom metal frame manufactured by HerbScan Engineering (London, U.K.). A scanned image is required to have, in addition to a barcode, a scale with the herbarium abbreviation and standardized colour chart placed visibly in the area of the sheet. Each specimen was digitized according to the following specifications: resolution: 600 pixels per inch (ppi); colour space: Adobe RGB (1998); colour depth: 24-bit; file format: uncompressed TIFF files; layout: portrait. Data about the specimens have been stored into the DATAflos (http://dataflos.sav.sk) database via internal software developed to manage Nábělek’s database. The stored data include specimen name, barcode, type status, collectors, collection number, locality description, locality altitude, and name revision history.

## Web portal

Digitized images of Nábělek’s Iter Turcico-Persicum collections at SAV are available via JSTOR Global Plants (https://plants.jstor.org/) and Biological Collection Access Service (http://www.biocase.org/). Nevertheless, in order to enhance the use of the digitized collection, we created a special web portal that not only presents the specimens, but also digitized publications by Nábělek, and last, but not least, enables annotations of specimens (the portal is available at www.nabelek.sav.sk).

The metadata on specimens are stored in a PostgreSQL (https://www.postgresql.org) database. The portal is a web application created in PHP (http://php.net) scripting language. It utilizes CakePHP 2.6.5 framework (http://api.cakephp.org/2.6/) to manage database connection, requests, their correct execution, and presentation of the results. It runs on Apache HTTP server version 2.4.7 (http://httpd.apache.org/docs/2.4/) on Ubuntu 14.04 (http://releases.ubuntu.com/14.04/). The presentation part uses HTML5 (https://www.w3.org/TR/html5/), CSS (https://www.w3.org/Style/CSS/), JavaScript’s jQuery library (http://jquery.com/), design and mobile friendliness is provided by the Bootstrap framework (http://getbootstrap.com/).

To search for a record, the user is presented with a quick search field offering a few choices to aim the search term - taxon name, taxon authors, collection number, specimen barcode, or any text (e.g. a word contained in locality description). In addition, a link ‘Search for specimens’ in the main menu leads to a page with search fields for the genus, species (both original determination and revisions), collection number, locality, and geographic coordinates with the possibility of specifying a range.

The detail page of a record displays all available information from the database, images associated with the record, and buttons for viewing or downloading the image. Detailed information on specimens includes the name of the plant according to the most recent identification, barcode number, collector name (mostly F. Nábělek), collection number of Iter Turcico-Persicum, herbarium in which specimen is deposited (SAV in all cases here, but in the future and with permission duplicates deposited in other herbaria will be added). In addition to this information, a CETAF stable identifier for each specimen (2016) [HTTP-URI-based persistent and stable identifiers for physical collection objects, http://cetaf.org/cetaf-stable-identifiers; Güntsch and Hagedorn 2013] is presented, to enable subsequent correct reference to the image or data. Locality data includes classification into the Level 1-4 of the World Geographical Scheme for Recording Plant Distributions (Brummitt 2001), and the verbatim detailed description as given on the specimen and in Nábělek’s publications. This information is mostly identical on labels and in publications. Locality data was completed by adding geographical coordinates. The revision history of the specimen includes original identification as published in Nábělek’s papers and subsequent revisions as found on annotation labels on specimens.

The detail page of a record includes also an interactive image viewer that allows user to zoom-in, zoom-out, or view image in its full resolution without needing to download the whole image. It is provided by IIPImage (About. http://iipimage.sourceforge.net). Providing the record has geographic coordinates, the locality is also shown on the minimap included on the detail page of each record. A possibility to post an annotation or updated determination to a record is provided to users. The process of annotation is managed by the AnnoSys facility (https://annosys.bgbm.fu-berlin.de/; Tschöpe et al. 2013).

The page “Map of all localities” presents a Google Map populated with all localities where the specimens were collected for which we have exact geographical coordinates. Each locality can be a place of collection of several specimens. Clicking the marker show the list of records. Overall, there are 2454 records that have been georeferenced.

On the page “Nábělek’s papers” preliminary digital versions of Nábělek’s papers are available for download. As soon as final digital copies are available in the Biodiversity Heritage Library (BHL; where their digitisation is currently ongoing), they will be replaced by the links to the material in BHL.

It is possible for visitor to comment on functionality and to suggest improvements via the project’s bugzilla page at Bugzilla: Nabelek herbarium (http://dataflos.sav.sk:8080/bugzilla/describecomponents.cgi?product=Nabelek%20herbarium).

The Nábělek website will be improved by introducing additional functionalities in future. All capabilities of the web interface as described here are present and functional in its version 1.0.

## Importance of the collection for local floras

Local and regional floras benefited from Nábělek’s collections, because at the time of his expedition there were no up-to-date accounts of the flora of the regions he visited. Boissier’s *Flora Orientalis* was already becoming out of date, having been published some 35 years earlier, and there were no contemporary Floras of Turkey, Palestine, Iraq or Iran. Moreover, the large number of new species that he had discovered, especially from the border areas of Iraq, Iran and Turkey, meant that his catalogue remained of major importance to workers in the region for the next fifty or so years. In particular, Peter H. Davis’s *Flora of Turkey and the East Aegean Islands* (Davis 1965–1985, Davis et al. 1988) was heavily dependent on Nábělek’s records from C9 Hakkari, as Peter Davis himself was the only other collector to have explored the flora of the Cilo Dağ region. Following Nábělek’s visit, the next significant botanical work in the area was carried out during the peregrination of Russian and Soviet botanists into the easternmost provinces of the former Ottoman Empire. Some of their records were mapped in Grossheim’s *Flora Kavkaza* (1928–1934, 1939–1967).
